# REVISITING THE US TEACHING NURSING HOME: WHAT ARE STUDENTS’ PERCEPTIONS OF CARING FOR OLDER ADULTS?

**DOI:** 10.1093/geroni/igae098.0714

**Published:** 2024-12-31

**Authors:** Erin Kitt-Lewis, Felicia Heaton, Maria Yoder, Donna Fick, Marie Boltz

**Affiliations:** The Pennsylvania State University, University Park, Pennsylvania, United States; Centre Care Rehabilitation and Wellness Services, Bellefonte, Pennsylvania, United States; The Pennsylvania State University, University Park, Pennsylvania, United States; The Pennsylvania State University, University Park, Pennsylvania, United States; The Pennsylvania State University, University Park, Pennsylvania, United States

## Abstract

The U.S. Census Bureau predicts that by 2050, over 20% of the U.S. population will fall into the category of older adults and will be the “core business” of the nursing profession. This growing demand is coupled with critical staff shortages, especially in nursing homes, a deficit that was exacerbated by the experiences of the COVID-19 pandemic. The American Association of Colleges of Nursing has identified care for older adults as a priority for undergraduate nursing programs and nursing education literature suggests there are critical barriers toward greater integration of the knowledge, skills, and dispositions needed to adequately care for older adults living in nursing homes. The purpose of this study was to understand undergraduate nursing students’ perceptions about their first clinical learning experience in a nursing home and working with older adults., Students (N= 105) from a large research university who were enrolled in the Nursing Fundamentals course were recruited to participate in the study. De-identified course artifacts (e.g., Age-Friendly Health Systems 4Ms Framework course assignments) generated by consented participants were compiled and analyzed using thematic analysis. Two themes emerged from the data and included students’ assumptions and lessons learned about caring for older adults living in nursing homes. In addition, students identified opportunities that could improve care for older adults, especially around “What Matters” and functional mobility. These findings will serve as the basis to guide best practices for integrating additional learning experiences in the undergraduate nursing curriculum using the Age-Friendly Health Systems 4Ms Framework.

